# Using Visualized Matrix Effects to Develop and Improve LC-MS/MS Bioanalytical Methods, Taking TRAM-34 as an Example

**DOI:** 10.1371/journal.pone.0118818

**Published:** 2015-04-24

**Authors:** Jia-Hung Ye, Li-Heng Pao

**Affiliations:** 1 Graduate Institute of Life Sciences, National Defense Medical Center, Taipei, Taiwan, Republic of China; 2 School of Pharmacy, National Defense Medical Center, Taipei, Taiwan, Republic of China; 3 Research Center for Industry of Human Ecology, Chang Gung University of Science and Technology, Kweishan, Taoyuan, Taiwan, Republic of China; 4 Graduate Institute of Health-Industry Technology, Chang Gung University of Science and Technology, Kweishan, Taoyuan, Taiwan, Republic of China; Moffitt Cancer Center, UNITED STATES

## Abstract

Matrix effects (MEs) continue to be an obstacle in the development of the LC-MS/MS method, with phospholipids being the major cause of MEs. Changing the mobile phase has been a common strategy to reduce MEs; however, the underlying mechanism is unclear. "In-source multiple-reaction monitoring" (IS-MRM) for glycerophosphocholines (PCs) has been commonly applied in many bioanalytical methods. "Visualized MEs" is a suitable term to describe the application of IS-MRM to visualize the elution pattern of phospholipids. We selected a real case to discuss the relationship of MEs and phospholipids in different mobile phases by quantitative, qualitative, and visualized MEs in LC-MS/MS bioanalysis. The application of visualized MEs not only predicts the ion-suppression zone but also helps in selecting an appropriate (1) mobile phase, (2) column, (3) needle wash solvent for the residue of analyte and phospholipids, and (4) evaluates the clean-up efficiency of sample preparation. The TRAM-34 LC-MS/MS method, improved by using visualized MEs, was shown to be a precise and accurate analytical method. All data indicated that the use of visualized MEs indeed provided useful information about the LC-MS/MS method development and improvement. In this study, an integrative approach for the qualitative, quantitative, and visualized MEs was used to decipher the complexity of MEs.

## Introduction

Liquid chromatography-tandem mass spectrometry (LC-MS/MS) is a versatile technique in the field of life sciences. Its high sensitivity, specificity, accuracy, and robustness help in qualifying and quantifying the analytes in a biological matrix. However, matrix effects (MEs), resulting from coeluting matrix components, lead to unreliable results [1]. The MEs are compound-dependent, and may enhance or suppress the ion in the LC-MS/MS analytical method [2]. Inadequate chromatography causes ion suppression and irreproducible results in some bioanalytical methods [3]. Electrospray ionization (ESI) is more prone to ion suppression than atmospheric pressure chemical ionization (APCI) [4]. It is important to evaluate the MEs in the method development, and they should be thoroughly assessed [5]. There are two strategies to determine MEs in an analytical method: qualitative evaluation (postcolumn infusion method) and quantitative evaluation (postextraction spike method) [6–9]. The postcolumn infusion method provides a qualitative assessment and an ion-suppression zone for analyte. The postextraction spike method is a quantitative assessment of MEs and is widely used for their evaluation in bioanalytical methods.

**Fig 1:**
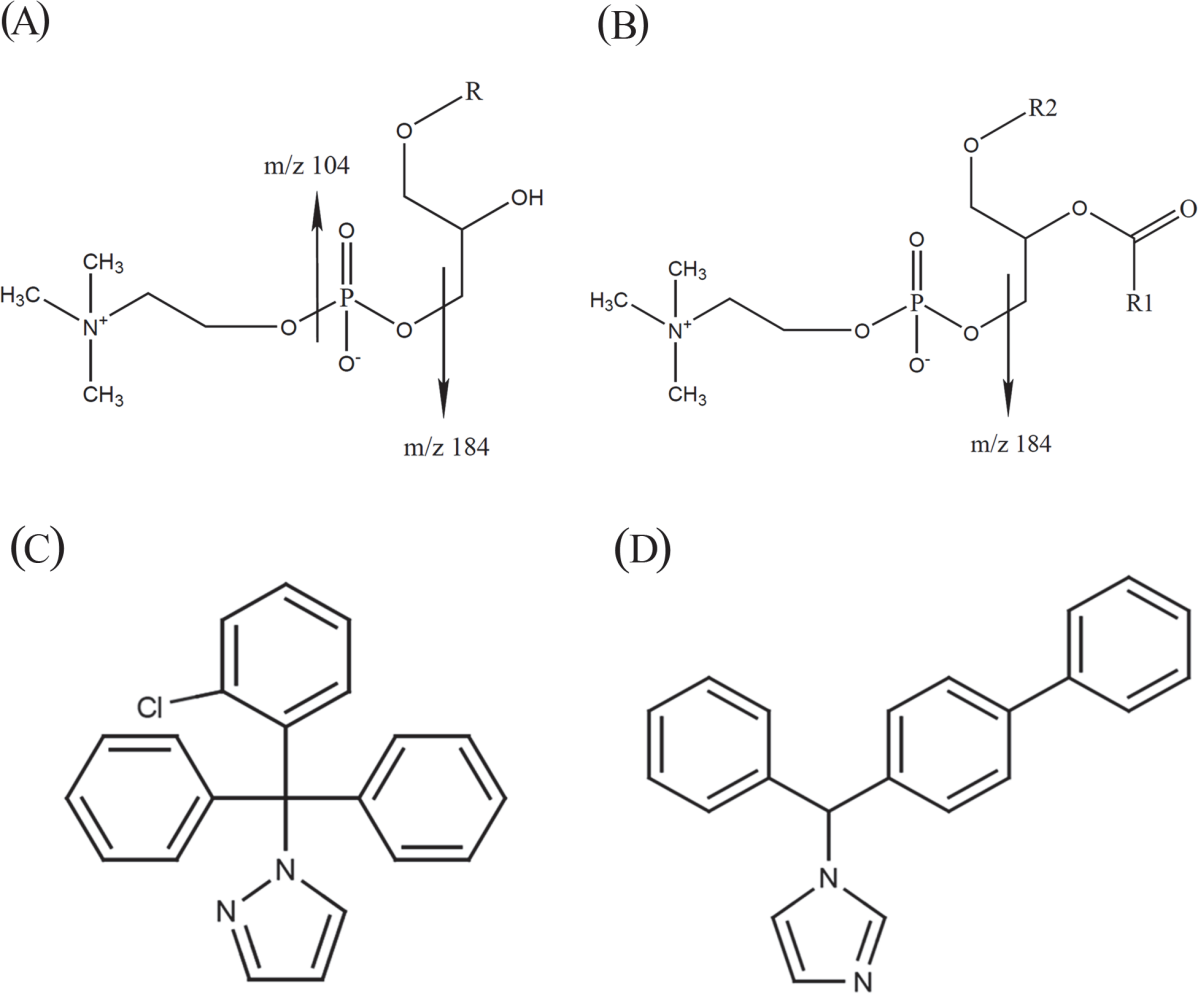
Structure of glycerophosphocholine (PC), lyso-PC (LPC), TRAM-34 and bifonazole. (A) Structure of LPC. The ion fragments are *m/z* 184 and *m/z* 104. (B) Structure of PC. The ion fragment is *m/z* 184. (C) Structure of TRAM-34. MW = 344. (D) Structure of bifonazole. MW = 311.

Phospholipids are the main components of the cell membranes and the major cause of MEs in bioanalytical methods [5, 10]. Phosphoglycerides is a source of phospholipids. The glycerophosphocholines (GPCho’s or PCs) are the major class of phospholipids in the plasma [11]. PCs can be divided according to their different glycerin groups into 1-mono (2-lyso) (Fig 1A) and 1,2-disubstituted (diradyl) PCs (Fig 1B) [5]. In-source collision-induced dissociation (CID) of PCs provided a convenient way to monitor PCs in the plasma [5]. The in-source CID properties of 2-lyso and diradyl PCs were different. The dominant in-source CID fragments of 2-lyso PC (LPC) were *m/z* 104 and 184, and that of diradyl PC was *m/z* 184 [5]. The multiple-reaction monitoring (MRM) transition (*m/z* 104 → 104) was used to visualize the elution of LPC. Because the *m/z* 184 → 184 transition could estimate the elution of both 2-lyso and diradyl PCs, it has been more commonly selected to monitor the elution of PCs. Monitoring all PCs in one transition was the main advantage of the “in-source multiple-reaction monitoring” (IS-MRM), which has been commonly applied in many bioanalytical methods [5, 6, 10, 12–16]. IS-MRM is very useful to monitor the retention time (RT) of the analyte and the coeluting phospholipids during method development.

“Visualized MEs” is a suitable term to describe the application of IS-MRM to visualize the elution pattern of PC, LPC, and SM in the chromatogram. Many applications have been developed for the IS-MRM transition to monitor the elution of phospholipids. This study describes the integrative view of the qualitative, quantitative, and visualized MEs. A highly selective K_Ca_3.1 channel blocker, 1-[(2-chlorophenyl) diphenylmethyl]-1*H*-pyrazole (TRAM-34) (Fig 1C), was derived from clotrimazole, and both of them share the same triphenyl moiety. Structurally, TRAM-34 has a pyrazole ring, and clotrimazole has an imidazole ring. K_Ca_3.1 plays a critical role in various physiological and pathological conditions such as vascular inflammation, atherogenesis, angiogenesis, renal fibrosis, cell migration, and proliferation of several kinds of cells [17–22]. TRAM-34 is the best candidate to investigate the importance of K_Ca_3.1 both *in vitro* and *in vivo*. We previously developed an LC-MS/MS method for the detection of TRAM-34 in the rat plasma using solid-phase extraction (SPE) [23]. However, significant MEs were observed when the TRAM-34 concentration was low. This motivated our further investigation into MEs. In this study, TRAM-34 was used to decipher the complexity of MEs by comprehensively discussing three kinds of MEs under different LC conditions.

MEs present obstacles in the LC-MS/MS method development. Changing the mobile phase is a common strategy to attenuate MEs; however, the underlying mechanism remains unclear. In this study, three different mobile-phase B solutions (acetonitrile (ACN), methanol (MeOH), and MeOH/ACN 50/50) were used for their different elution properties for phospholipids and TRAM-34. First, we used the same SPE method and LC gradient elution profile with three different mobile-phase B solutions to investigate the relationship between TRAM-34, phospholipids, and MEs. Second, we used visualized MEs to improve the TRAM-34 method and the sample preparation method was changed from SPE to protein precipitation (PPT). Third, we shortened the gradient elution time from 7.5 min to 6 min with the help of visualized MEs. Finally, we evaluated the linearity, accuracy, precision, and qualitative MEs to verify the feasibility of the visualized MEs. Our integrated approach for qualitative, quantitative, and visualized MEs provided an appropriate model to unravel the relation between the MEs and the changing mobile phase.

## Material and Methods

### Materials

TRAM-34 was purchased from Toronto Research Chemicals (Ontario, Canada). Bifonazole (Fig 1D, IS) and formic acid (FA) were purchased from Sigma-Aldrich (St. Louis, MO, USA). ACN, MeOH, and isopropanol (IPA) were of LC-MS grade and were purchased from JT Baker (Philipsburg, NJ, USA). Water was prepared using a Millipore Direct Q3 water purification system (Millipore, Billerica, USA).

### Plasma

Six male nine-week-old Sprague–Dawley (SD) rats (BioLasco Taiwan Co., Ltd., Taipei, Taiwan) were used with approval from the Institutional Animal Care and Use Committee (IACUC) of National Defense Medical Center (NDMC) (IACUC-12-254). The housing, care, diet, and maintenance of the experimental animals were consistent with the recommendations of the National Research Council’s Guide for the Care and Use of Laboratory Animals, Animal Welfare Act, and NDMC Policy. The SD rats were 315 ± 10 g at nine weeks; they were anesthetized by Zoletil 50 (Carros Cedex, France) before performing the carotid artery catheterization. A blood sample was collected into a heparin-containing tube. The plasma was collected after the blood sample had been centrifuged at 12000 rpm for 10 min. These plasma samples were stored at −80°C.

### Preparation of standard and quality-control samples

The concentration of the TRAM-34 stock solution was 1 mg/mL in MeOH. The concentrations of the working standards were in the range 10 ng/mL–10 μg/mL; they were prepared by serial dilutions with MeOH. The concentration of the IS stock solution was 1 mg/mL; it was diluted to 0.2 μg/mL with 50% MeOH in water. Seven plasma calibration standards and three quality control (QC) samples were prepared by spiking the TRAM-34 working standard into the rat blank plasma. The final concentrations of the seven calibration samples were 1, 10, 100, 250, 500, 700, and 1000 ng/mL. Seven plasma calibration standards and three QC samples were prepared by spiking 10 μL of the TRAM-34 working standard into 90 μL of the rat blank plasma. Then, 100 μL of a 0.2 μg/mL IS solution was added into the TRAM-34-spiked rat plasma to yield the final 200 μL samples. The three QC samples were 3 ng/mL (low-concentration QC, LQC), 450 ng/mL (medium-concentration QC, MQC), and 800 ng/mL (high-concentration QC, HQC).

### Sample preparation

#### 1. SPE method

SPE was performed to extract TRAM-34 and bifonazole from the rat plasma [23]. The Strata-X 30 mg/mL SPE column (Phenomenex, Torrance, USA) was conditioned and equilibrated with ACN and water. The plasma sample solution (200μL) was loaded into the SPE cartridge, which was then washed with 40% MeOH and eluted with ACN, respectively. The eluted samples were evaporated and dried under a stream of nitrogen at 45°C for 10 min using a Caliper TurboVap LV apparatus (Zymark, Portland, USA). A 100 μL aliquot of the reconstitution solution, ACN/water (1:1 v/v), was added into each of the dried samples. These reconstituted samples were then transferred to the inserts of the autosampler vial for LC-MS/MS analysis.

#### 2. PPT method

The PPT method was performed to extract TRAM-34 and IS from the rat plasma. One hundred and fifty μL of the 0.167-μg/mL IS solution (in ACN) was added into the TRAM-34-spiked rat plasma to yield the final 200-μL samples. These calibration standards and QC samples were centrifuged at 12,000 rpm and 4°C for 10 min. Finally, 150 μL of the supernatant was transferred to the inserts of the autosampler vial for LC-MS/MS analysis.

### Liquid chromatography-tandem mass spectrometry conditions

The liquid chromatography (LC) system used was the Agilent 1200 series (Agilent Technologies, Santa Clara, USA). An XBridge BEH phenyl column (50 × 3 mm, 5 μm particle size, Waters, Milford, USA) and SecurityGuard phenyl guard cartridges (4 × 2.0 mm, Phenomenex, Torrance, USA) were used to perform the separation. The column oven temperature was 50°C.

The LC system was equipped with a triple quadrupole API 3200 LC-MS/MS system (AB Sciex, MA, USA). The Turbo V ion source of API 3200 was operated in positive-mode electrospray ionization (ESI), and the ion-spray voltage was 5 kV. The temperature of the ion source was 500°C. According to the 2002/657/EC guidelines, one precursor ion and two product ions could meet the criteria of compound identification (identification points, IP = 4) [24]. The most abundant MRM transition was selected for quantification, and the second most abundant MRM transition was selected for qualification. Two MRM transitions were tuned for TRAM-34: *m/z* 277.2 → 165.1 (for quantification) and *m/z* 277.2 → 241.2 (for qualification). The detailed parameters of the MRM transition are listed as follow: TRAM-34, *m/z* 277.2 → 165.1, DP = 48, CE = 38.7; TRAM-34, *m/z* 277.2 → 241.2, DP = 12.9, CE = 20.3; bifonazole, *m/z* 311.3 → 243.1, DP = 26, CE = 37.9; PC, *m/z* 184.1 → 184.1, DP = 121.6, CE = 7; LPC, *m/z* 104.1 → 104.1, DP = 105.0, CE = 6. Data acquisition and quantification were performed using Analyst^TM^ 1.4.2 software.

### LC method

#### 1. For SPE samples

The reconstituted samples (processed by SPE) were transferred to the inserts of the autosampler vial for LC-MS/MS analysis. The LC conditions were as follows: mobile phase A, was 0.2% FA in water; mobile phase B, 0.2% FA in ACN/MeOH (1:1 v/v), 0.2% FA in MeOH, or 0.2% FA in ACN. The flow rate was 400 μL/min. The gradient elution was as follows: mobile phase A: 0–1 min, 70–30%; 1–1.5 min, 30–0%; 1.5–4.5 min, 0%; and 4.51–7.5 min, 70%. The run time of a single injection was 7.5 min. The autosampler temperature was 4°C. The sample injection volume was 5 μL.

#### 2. For PPT samples

The reconstituted samples (processed by PPT) were transferred to the inserts of the autosampler vial for LC-MS/MS analysis. Mobile phase A was 0.2% FA in water, and mobile phase B was 0.2% FA in ACN. The flow rate was 600 μL/min. The gradient elution was as follows: mobile phase A: 0–1 min, 70–30%; 1–1.5 min, 30–0%; 1.5–3.5 min, 0%; and 3.51–6 min, 70%. The run time of a single injection was 6 min. The autosampler temperature was 4°C. The sample injection volume was 10 μL.

### Identification of tentative phospholipids

The Q1 full scan (*m/z* 400–1000) was performed to record the spectrum of the double blank sample (SPE-processed) under the LC-MS/MS conditions. By using the commercial software MarkerView version 1.2.1 and LipidView version 1.2 (AB Sciex, MA, USA), it was easier to process the data and identify the phospholipid candidates. The raw data were imported into LipidView, and the polarity was positive, the minimum signal to noise (S/N) ratio was 5, and the analysis types were glycerophospholipids (containing LPCs and PCs) and sphingolipids (SMs). This analysis was selectively focused on specific phospholipid classes and that other lipids can be presented in the range where LPCs, PCs and SMs were present. Those unidentified candidates might be other species of lipids, steroids, or endogenous metabolites.

### Evaluation of qualitative matrix effects

The postcolumn infusion experiment was achieved by the constant infusion of 1000 ng/mL of TRAM-34 and 2000 ng/mL of IS. The Harvard infusion pump was used to infuse the TRAM-34 and IS solutions through a T-connector into the ion source at a speed of 10 μL/min. Under the constant infusion of the TRAM-34 solution, the chromatogram of TRAM-34 MRM transition (*m/z* 277.2 → 165.1) was compared between the injection of the SPE-extracted plasma and ACN/water (1:1 v/v) solution. Under the constant infusion of IS solution, the IS postcolumn infusion experiment was executed according to the above process. The chromatography provided a qualitative assessment of the MEs in the RT region of TRAM-34 and IS.

### Evaluation of quantitative matrix effects

The first set (Set 1) was defined as that containing TRAM-34 and IS obtained after spiking with the SPE-treated rat plasma. The second set (Set 2) was defined as that containing TRAM-34 and IS spiked with ACN/water (1:1 v/v) solution. There were six replicates of the QC samples in Set 1 and Set 2; the peak areas of TRAM-34 and IS were calculated for the quantitative MEs (Set 1/Set 2 × 100 (%)) [9].

### Improved TRAM-34 method

#### 1. Selectivity, linearity, and lower limit of quantification

Selectivity was evaluated by six rat blank plasma samples, collected from different sources. The lower limit of quantification (LLOQ) had an S/N ratio of >10. The linearity was assessed from the slope of seven nonzero points on the calibration curve. The weighting was 1/x. The linear range was 1–1000 ng/mL; the correlation coefficient was accepted when the value was >0.995.

#### 2. Accuracy and precision

The evaluation criteria of accuracy of the QC samples in the within-run and between-run tests was within ±15% range of the nominal concentration, while the criterion for the LLOQ was within ±20% of the nominal concentration. The precisions of the QC samples in the within-run and between-run tests were accepted when the coefficients of variation (CV) values were <15% (<20% for LLOQ) [25].

## Results and Discussion

### Visualizing phospholipid elution pattern by IS-MRM using SPE method

**Fig 2:**
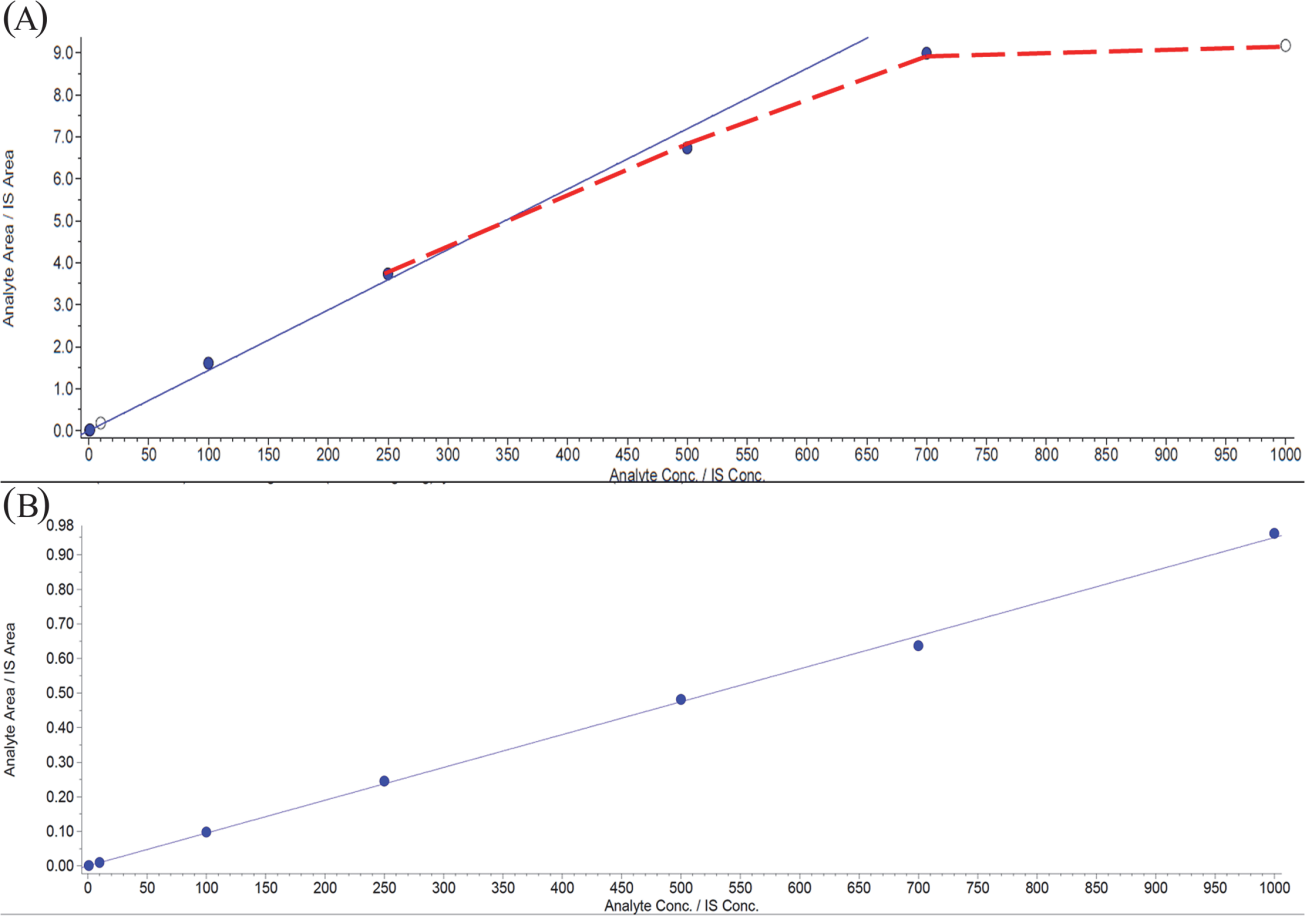
Plateaued calibration curve using MeOH + 0.2% FA, (A). Blue line represents the calibration curve; red line represents the plateau curve at high concentrations. calibration curve using ACN + 0.2% FA (r = 0.9996), (B). When comparing the linearity in Fig. 2A and 2B, it represents that there might be massive ion suppression in MeOH + 0.2% FA.

During the TRAM-34 LC-MS/MS method development with SPE-sample preparation, when mobile phase B was 0.2% FA in MeOH, the response between TRAM-34 and the internal standard (IS) showed a plateau in the curve (Fig 2A). Although SPE had the advantages of cleaning and concentrating the sample, apart from acting as a solvent exchange, the results still indicated that it could not avoid MEs. The use of 0.2% FA in ACN/MeOH (50/50) as a solvent for mobile phase B was found to resolve the problem of linearity; however, the LQC still showed massive ion suppression (46.1%) [23]. It is interesting to investigate the relation between the mobile phase and the linearity.

It was inferred that the plateau of the calibration curve did not result from saturated ionization. Initially, the signal was very high when we spiked the 50% MeOH solution with 1000 ng/mL TRAM-34. We then spiked the plasma with 1000 ng/mL TRAM-34, and the signal was significantly decreased. Finally, a plateau was observed in the curve when the number of injections was increased. The calibration curve without the influence of MEs in ACN + 0.2% FA showed good linearity (Fig 2B). The influence of the MEs on bioanalysis was considered and was the motivation for this study. The plateau in the curve (Fig 2A) demonstrated that there might be massive ion suppression. MEs are the major cause of ion suppression in LC-MS/MS analytical methods. It is rational to propose that the different mobile-phase solutions might affect the MEs. To verify this, the IS-MRMs for phospholipids were applied using this analytical method. By simultaneously monitoring the elution of phospholipids and analytes under different mobile-phase solutions, a new insight might be gained for improving the TRAM-34 LC-MS/MS analytical methods.

Because Little *et al*. demonstrated that IS-MRM is a good strategy to monitor the elution of phospholipids [5], the MRM transitions of *m/z* 104.1 → 104.1 and *m/z* 184.1 → 184.1 were tuned in our system (Figure A–B in S1 Fig). By applying two IS-MRM transitions in the TRAM-34 method development, it was interesting to visualize the elution time of TRAM-34 and the phospholipid elution pattern in the chromatograms.

**Fig 3:**
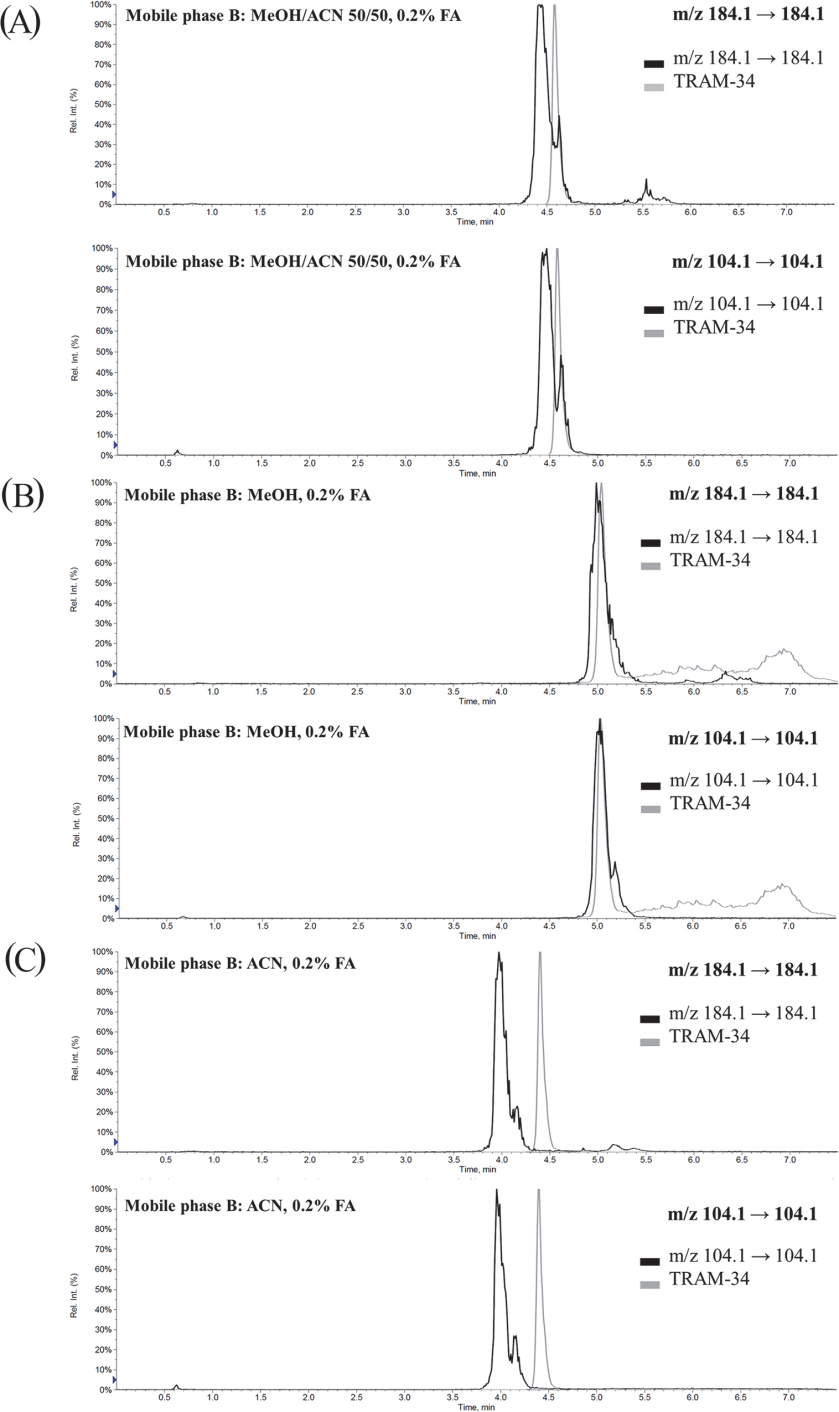
Visualized elution patterns of phospholipids and TRAM-34 in in-source multiple-reaction monitoring (IS-MRM) channels. (A) Mobile phase B is MeOH/ACN 50/50 + 0.2% FA. Relative elution time and elution pattern of TRAM-34 and phospholipids for both the *m/z* 184.1 → 184.1 and *m/z* 104.1 → 104.1 transitions. Elution of TRAM-34 was at the trough of that of the phospholipids. (B) Mobile phase B is MeOH + 0.2% FA. Relative elution time and elution pattern of TRAM-34 and phospholipids for both the *m/z* 184.1 → 184.1 and *m/z* 104.1 → 104.1 transitions. Elution of TRAM-34 was in the largest peak of phospholipids. (C) Mobile phase B is ACN + 0.2% FA. Relative elution time and elution pattern of TRAM-34 and phospholipids for both *m/z* 184.1 → 184.1 and *m/z* 104.1 → 104.1 transitions. Elution of TRAM-34 was almost separate from that of phospholipids.

When mobile phase B is MeOH/ACN 50/50 with 0.2% FA (MP-B1), the elution peak of TRAM-34 was located in the trough of the elution pattern of the phospholipids (Fig 3A). These results revealed that the intensity and/or ionization of TRAM-34 under these conditions might be affected by the coeluted phospholipids.

When using 100% MeOH with 0.2% FA (MP-B2), the elution patterns of TRAM-34 and the phospholipids overlapped (Fig 3B). Because phospholipids are the major cause of MEs in LC-MS/MS, the coelution of phospholipids is usually responsible for the massive ion suppression [15]. This might be the reason for the plateau in the curve (Fig 2A).

Judging from these observations, it was necessary to examine the elution pattern of TRAM-34 and phospholipids with 0.2% FA in 100% ACN (MP-B3). Surprisingly, the elution of TRAM-34 was nearly free from these phospholipids (Fig 3C). This exciting result provided new insight into the development and improvement of TRAM-34 LC-MS/MS analytical methods.

The elution patterns of IS and the phospholipids in the three mobile-phase B solutions (MP-B1–B3) were monitored (S2 Fig); no visible coelution interference for IS in the IS-MRM channels was observed. A comparison of the elution patterns of the phospholipids in the chromatograms of *m/z* 184.1 → 184.1 revealed that the three mobile-phase B solutions had different effects on the elution of the phospholipids using the same LC equipment.

In summary, these observation in the three different mobile-phase B solutions demonstrated that the visualization of the phospholipid elution pattern might be of tremendous benefit to LC-MS/MS method development and improvement.

### Evaluation of qualitative matrix effects by postcolumn infusion

**Fig 4:**
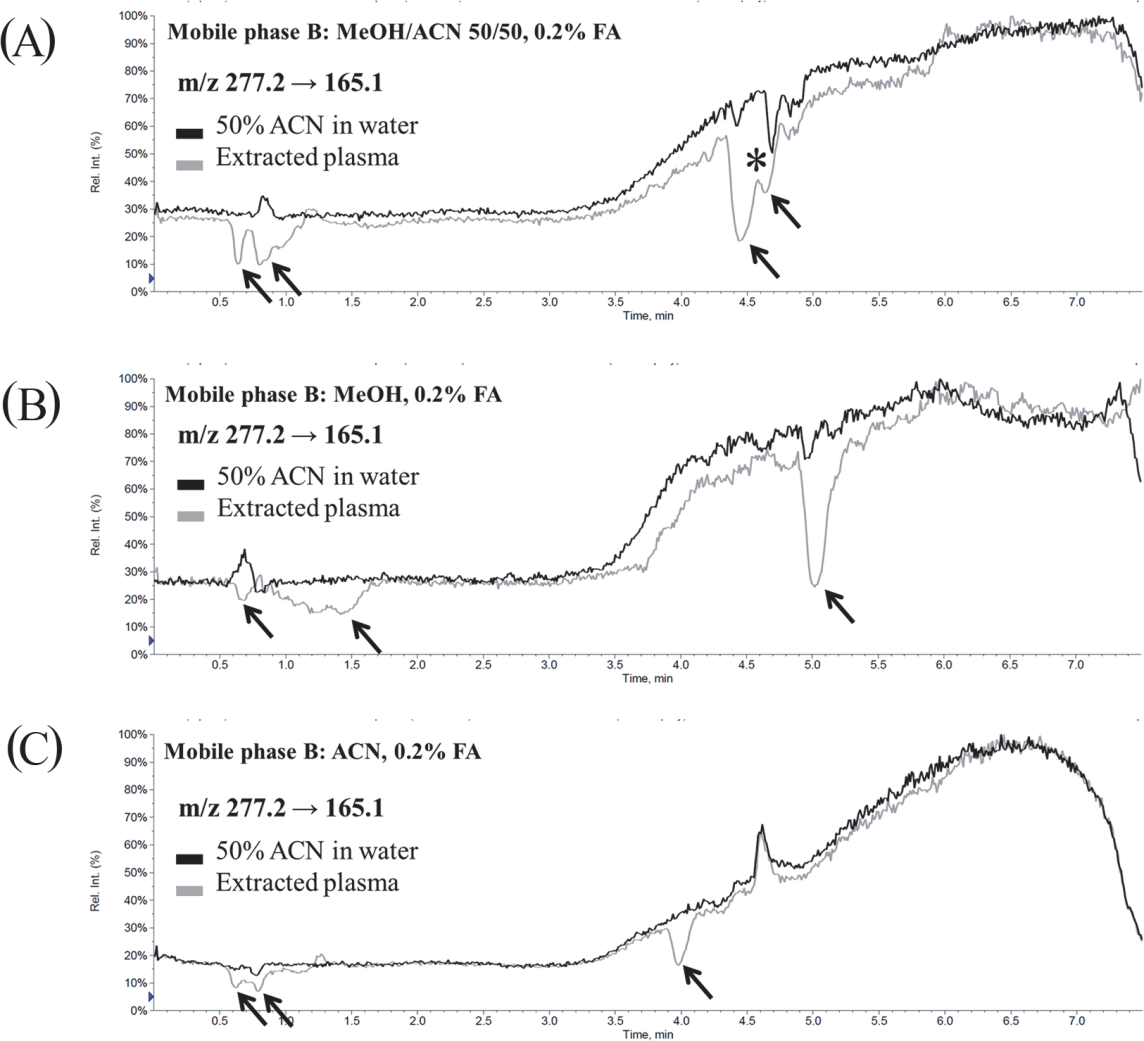
Qualitative matrix effects (assessment by postcolumn infusion) for TRAM-34. (A) Mobile phase B is MeOH/ACN 50/50 + 0.2% FA. There were four dips that represent ion suppression in the chromatogram. Asterisk: elution of TRAM-34 was located in the peak of ion-suppression zone. (B) Mobile phase B is MeOH + 0.2% FA. There were three dips that represent ion suppression in the chromatogram. Elution of TRAM-34 was located in the largest dip. (C) Mobile phase B is ACN + 0.2% FA. There were three dips that represent ion suppression in the chromatogram. Elution of TRAM-34 was not located in the dip.

Postcolumn infusion is a common strategy to access the ion-suppression zone, which is affected by coeluting compounds, in an LC-MS/MS method. In order to verify whether the ion suppression results from the visualized patterns in the IS-MRM channels, postcolumn infusion experiments were performed. The continuous infusion of TRAM-34, the dip, compared to the injection of 50% ACN in water, represented the ion-suppression zone of TRAM-34 in the LC-MS/MS method. In the chromatogram of MP-B1 (Fig 4A), four distinctive dips (marked with arrows) were observed. The qualitative ME data were compared to the corresponding visualized IS-MRM data; the dips in the RT between 4.0 and 5.0 min were found to be consistent with the elution patterns of the phospholipids. The elution of TRAM-34 was located in the trough of the phospholipids (Fig 3A) and in the peak of the ion-suppression zone (Fig 4A, asterisk). These data indicate that IS-MRM could predict the ion-suppression zone in an LC-MS/MS method.

There were three dips (arrows) in the chromatogram of MP-B2 (Fig 4B). The elution of TRAM-34 and the phospholipids in Fig 3B and the biggest dip in Fig 3B are consistent, which explains the plateau in the curve (Fig 2A). Because of the competition of ionization, the response of TRAM-34 is suppressed by the phospholipids.

Similarly, there are three dips (arrows) in the chromatogram of MP-B3 (Fig 4C). By comparing Fig 3C and Fig 4C, it is reasonable to assume that TRAM-34 might experience the smallest MEs in the MP-B3 mobile phase. All the data in the visualized phospholipid elution pattern and qualitative MEs show that elution of phospholipids will cause massive ion suppression.

The postcolumn infusion data of IS are presented in S3 Fig The dips in the chromatograms are marked with arrows. A comparison of the data in S2 Fig and S3 Fig suggested that the phospholipids were responsible for the ion suppression. The continuous infusion of TRAM-34 or IS showed a different response to the elution of phospholipids, which resulted in a different number of dips (when using MP-B1). The difference between the qualitative MEs of TRAM-34 and IS revealed that the MEs were compound-dependent.

Interestingly, the same ion-suppression zone during the RT 0.5–1.5 min was observed in all the chromatograms of the qualitative MEs. In the early-elution zone, the ion suppression has been regarded as the product of salts, other nonretained components, and polar proteins and carbohydrates [3, 5]. In this study, the transitions of *m/z* 184.1 → 184.1 and *m/z* 104.1 → 104.1 could briefly explain the ion suppression in the early-elution zone (S4 Fig). The coincidence of the peaks present in the *m/z* 184.1 → 184.1 and *m/z* 104.1 → 104.1 transitions and the dips observed in the postcolumn infusion chromatograms suggested that the phospholipids might be responsible for the ion suppression in the early elution. In addition, the issue of dead volume could occur at this time, and may be a factor for ion suppression during the RT of 0.5–1.5 min.

Given the elution patterns of the phospholipids in the IS-MRM chromatograms and the dips in the qualitative MEs, the visualized elution patterns of the phospholipids could predict the ion-suppression zone in the LC-MS/MS method, thereby providing useful information for the development of LC-MS/MS analytical methods.

### Evaluation of quantitative matrix effects

As mentioned above, MEs resulted from the different mobile-phase B solutions influenced the linearity during the TRAM-34 analytical method development. A comparison of the chromatograms of the visualized MEs shows that qualitative MEs and quantitative MEs in three different mobile-phase B solutions could reveal the importance of the relationship of MEs with phospholipids in LC-MS/MS bioanalysis. To investigate the relation between the mobile-phase B solutions and the quantitative MEs, MP-B1, MP-B2, and MP-B3 were used to evaluate the quantitative MEs. The data of the quantitative MEs in the three different mobile-phase B solutions are listed in Table 1. The quantitative MEs of LQC, MQC, and HQC in MP-B1 were 46.1±3.8%, 74.6±6.0%, and 73.8±8.7%, respectively [23]. The MEs of LQC, MQC, and HQC in MP-B2 were 33.3±2.7%, 64.4±3.2%, and 62.6±1.9%, respectively. These results demonstrated that the elution of TRAM-34 in the intensive region or trough region of the coeluting phospholipids influenced the quantitative MEs. In the presence of phospholipids, LQC suffered massive ion suppression, while MQC and HQC showed minor ion suppression.

**Table 1:** The Matrix effects data (mean ± s.d.).

		ACN/MeOH 1:1 solution B (n = 6)		100% MeOH solution B (n = 6)			100% ACN solution B (n = 6)	
Nominal concentration (ng/ml)		Calculated matrix effect (%)		Calculated matrix effect (%)			Calculated matrix effect (%)	
		TRAM-34	Bifonazole	TRAM-34		Bifonazole	TRAM-34	Bifonazole
3		46.1 ± 3.8	86.5 ± 3.9	33.3 ± 2.7		90.3 ± 2.1	113.9 ± 7.7	85.0 ± 3.9
450		74.6 ± 6.0	88.1 ± 5.5	64.4 ± 3.2		101.7 ± 7.6	110.1 ± 1.2	89.2 ± 2.6
800		73.8 ± 8.7	88.0 ± 5.2	62.6 ± 1.9		105.0 ± 3.1	106.7 ± 2.0	91.4 ± 2.3

The MEs of LQC, MQC, and HQC in MP-B3 were 113.9±7.7%, 110.1±1.2%, and 106.7±2.0%, respectively. It was interesting to compare the qualitative (Fig 4C) and quantitative MEs with the visualized phospholipid-elution patterns (Fig 3C). Using MP-B3, the elution of TRAM-34 was almost separate from that of the phospholipids (Fig 3C); the qualitative and quantitative MEs (Fig 4C and Table 1) showed that TRAM-34 experienced nearly no MEs. In summary, the visualized phospholipids elution pattern may be of tremendous benefit for LC-MS/MS analytical method development and improvement as a time-saving and economic strategy to assess and predict qualitative and quantitative MEs.

Visualized MEs is a suitable term to describe the application of IS-MRM to visualize the elution pattern of PCs, LPCs, and SMs. We also examined a real case to discuss the relationship of the MEs with phospholipids according to the quantitative, qualitative, and visualized MEs in the LC-MS/MS bioanalysis. The underlying mechanism of the change of solvent was revealed by performing an integrative examination of these three kinds of MEs. Different solvents possess various elution abilities for the analyte and phospholipids, which results in different MEs during LC-MS/MS analysis.

### Identification of tentative phospholipids causing ion suppression

In LC-MS/MS, LPCs are more likely to produce MEs [15]. To investigate which phospholipids caused the massive ion suppression, a Q1 full scan and a precursor ion scan of *m/z* 184.1 were performed. MP-B1 was used to investigate the candidate phospholipids in the ion-suppression zone. The data were further processed by LipidView and MarkerView software to identify the possible phospholipid species. There were 102 species of phospholipids identified by the software in the Q1 scan chromatogram (S5 Fig). The part of the chromatogram between the RTs of 4.0 and 5.0 min in the Q1 full scan was extracted to investigate the tentative phospholipids that resulted in the massive ion suppression (S6 Fig). Five tentative phospholipids (*m/z* 496.5 = LPC 16:0, *m/z* 520.5 = LPC 18:2, *m/z* 524.5 = LPC 18:0, *m/z* 542.4 = LPC 20:5, and *m/z* 747.8 = SM 36:1;3) were identified by LipidView at RT 4.0–5.0 min that may cause the massive ion suppression for TRAM-34 (Fig. A in S7 Fig). Four of these identified tentative phospholipids were LPCs.

The *m/z* 184.1 → 184.1 and *m/z* 104.1 → 104.1 transitions could be used to distinguish the LPCs from PCs [15]. Accordingly, the presence of phospholipids in two IS-MRM channels and the identified tentative LPCs constituted strong evidence that LPCs were the major species of phospholipids causing MEs in this study. Two of the five tentative phospholipids candidates, *m/z* 496.5 = LPC 16:0 and *m/z* 524.5 = LPC 18:0, were previously reported to be early-eluted phospholipids in LC-MS/MS analysis [16].

A precursor ion scan of *m/z* 184.1 was also performed to investigate whether phospholipids are the only source of MEs at RT 4.0–5.0 min. The Q1 full scan showed more candidates causing the MEs than the precursor ion scan of *m/z* 184.1 (Fig. B in S7 Fig). This analysis was selectively focused on specific phospholipid classes and that other lipids can be presented in the range where LPCs, PCs and SMs were present. Those unidentified candidates might be other species of lipids, steroids, or endogenous metabolites. In addition, the low resolution MS in this study had the limits to identify the tentative phospholipids as well as the isobaric compounds. Further investigation is needed to figure out the relationship between these candidates and the MEs.

### Improving TRAM-34 LC-MS/MS analytical method by visualized matrix effects

**Fig 5:**
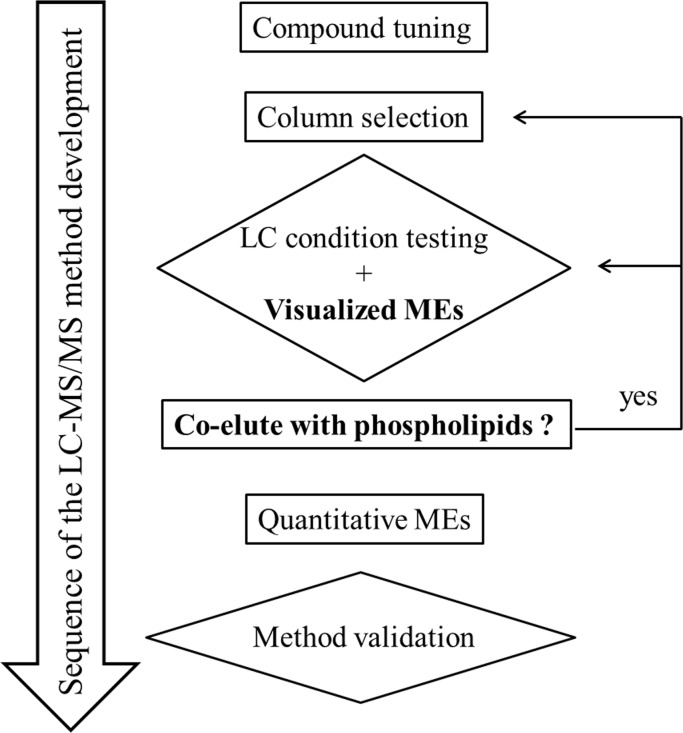
Flowchart of LC-MS/MS method development. During LC-MS/MS method development, compound tuning, column selection, LC condition testing, method validation, and quantitative matrix effects assessment are necessary. To boost LC-MS/MS method development, use of visualized matrix effects is introduced. By monitoring the elution of the analyte and phospholipids, it is easier to evaluate whether the LC conditions are appropriate. Visualization of the phospholipid elution pattern is of tremendous benefit to LC-MS/MS method development and improvement.

According to the above findings, the TRAM-34 LC-MS/MS method could be easily improved by employing the strategy of visualized MEs. Fig 5 shows the scheme to develop the improved TRAM-34 LC-MS/MS method in this study, which was also feasible for other LC-MS/MS analytical method development. The selection of the column was a critical issue in the development of LC-MS/MS method. The retention of the phospholipids varied with the different stationary phase. From the chromatogram of the visualized MEs in 100% ACN (Fig 3C), it can be inferred that TRAM-34 and phospholipids could be easily separated. The force of π-π interaction between the phenyl column and TRAM-34 may be stronger than the hydrophobic forces between the phospholipids and the phenyl column. The phospholipids showed the highest elution when the phenyl column was eluted with 100% ACN followed by 100% methanol and 50/50 ACN/MeOH. Therefore, a phenyl column with 100% ACN (mobile-phase B) was chosen to verify the feasibility of the visualized ME strategy in the present study. More importantly, gradient elution was more effective for the removal of phospholipids than isocratic elution. In fact, a high organic component (100%) of the mobile phase was most effective for the elution of phospholipids [15]. In the chromatograms of IS-MRM, the phospholipids were eluted by the 100% organic phase. The appropriate gradient elution and column selection in this study prevented the accumulation of phospholipids.

By introducing visualized MEs (Fig 5), it was easier to assess the adequacy of the column and the LC conditions. The visualized elution pattern of phospholipids could predict the ion-suppression zone according the above results. When the analyte coelutes with the phospholipids, changing the column or LC conditions should be seriously considered.

With the correlation of visualized, qualitative, and quantitative MEs revealed in the present study, it is easier to improve LC-MS/MS methods. We modified the LC conditions and changed the sample preparation method using the strategy of visualized MEs. Although SPE was usually thought to result in fewer MEs, the present data showed that most phospholipids were not removed by SPE under these experimental conditions. Instead of removing phospholipids, the different elution sequence of the analyte and phospholipids provided new insight into how one can mitigate the MEs. Although the PPT method was more economic and time-saving, it would result in more MEs than SPE for sample preparation in general. After the visualized MEs were used to make sure that the elution sequence of TRAM-34 and phospholipids was not affected by PPT, the sample preparation was changed from SPE to PPT.

The “LC for SPE method” and “LC for PPT method” were used as two different analytical methods, and the results were compared. In the “LC for PPT method,” the flow rate was increased, and the elution sequence of the phospholipids and TRAM-34 in MP-B3 was unchanged (S8 Fig). When the sample preparation method was changed from SPE to PPT, the visualized phospholipids elution patterns also changed (Fig 3C and S8 Fig). PPT resulted in numerous elution patterns after the elution time of TRAM-34 in the *m/z* 184.1 → 184.1 channel, but not in the *m/z* 104.1 → 104.1 channel. The simultaneous monitoring of the *m/z* 184.1 → 184.1 and *m/z* 104.1 → 104.1 transitions could distinguish the LPCs from the PCs [5, 15]. Those late-eluting components in the *m/z* 184.1 → 184.1 channel were regarded as the PCs [15]. After SPE processing, the elution of PCs was smaller than after PPT processing. These observations indicated that the PCs were efficiently removed by SPE, but the LPCs were not. The setting of IS-MRM was also useful to assess the extent of clean-up of the sample during preparation.

To simultaneously wash the residue of TRAM-34 and phospholipids in LC system, various needle wash solvents were used: ACN, MeOH, IPA, MeOH/ACN 1:1, ACN/IPA 1:1, and MeOH/IPA 1:1 (Figure A and B in S9 Fig). IPA was the best wash solvent for TRAM-34, and MeOH was the best solvent for phospholipids. Thus, MeOH/IPA 1:1 was selected as needle wash solvent to simultaneously wash the residue of TRAM-34 and phospholipids.

In summary, the TRAM-34 LC-MS/MS analytical method was improved by using the strategy of visualized MEs. The improved “LC for PPT method” is summarized as follows: sample volume, 45 μL; sample preparation, PPT; needle wash solvent, MeOH/IPA 1:1; run time, 6 min; and flow rate, 600 μL/min. The LLOQ was still 1 ng/mL. Further method validation is needed to verify the feasibility of the strategy of visualized MEs in method development.

### Evaluated the improved TRAM-34 LC-MS/MS analytical method by selectivity, linearity, LLOQ, accuracy and precision

#### 1. Evaluation of quantitative matrix effects in the improved TRAM-34 method

The data of the quantitative MEs in the “LC for PPT method” are listed in Table 2. The MEs in the LQC, MQC, and HQC samples using the “LC for PPT method” were 95.5±1.4%, 93.3±0.6%, and 95.4±0.6%, respectively. These data were compared to the quantitative MEs in Table 1. The difference represented the complexity of the MEs. Table 1 and Table 2 present data acquired under the same LC-MS/MS conditions except for the flow rate and gradient elution profile. The flow rate and gradient elution profile might contribute the difference of quantitative MEs between Table 1 and Table 2. Another difference was the sample preparation (SPE vs. PPT). All of these factors might result in the difference between the quantitative MEs in Table 1 and Table 2.

**Table 2:** The matrix effects data in the improved TRAM-34 method (mean ± s.d.).

	Matrix effect data (n = 6)	
Nominal concentration (ng/ml)	Calculated matrix effect (%)	
	TRAM-34	Bifonazole
3	95.5 ± 1.4	92.0 ± 1.7
450	93.3 ± 0.6	96.8 ± 0.3
800	95.4 ± 0.6	95.8 ± 0.5

Set 1 = TRAM-34 and IS spiked in the extracted plasma.

Set 2 = TRAM-34 and IS spiked in the acetonitrile:water.

Matrix effect = Set 1/ Set 2 × 100

There are many commercial products to eliminate or decrease the MEs, but their high cost is a major issue for most labs. This study shows that changing the mobile phase could effectively avoid the phospholipid-induced MEs, representing a simple, fast, and economical strategy.

In summary, the quantitative MEs in this study were showed that the phospholipids in the visualized MEs were responsible for the massive ion suppression.

There were nearly no MEs when the elution of TRAM-34 was separate from the elution pattern of the phospholipids.

#### 2. Selectivity, linearity, and lower limit of quantification

The selectivity was evaluated using six different rat plasma samples. Setting two MRM channels for the TRAM-34 provided good selectivity [23]. There was no cross-talk in the analysis. The LLOQ was 1 ng/mL. The weighting was 1/*x*. The linearity was excellent (r = 0.9989–1.00) (Fig 2B, r = 0.996).

#### 3. Accuracy and precision

The accuracy and precision within-run and between-run in the “LC for PPT method” are listed in Table 3. The within-run accuracy of the “LC for PPT method” was 96.2–104.7%. The within-run precision of the “LC for PPT method” was 1.2–6.9 (CV%). The within-run accuracy and precision of the “LC for PPT method” were better than in the “LC for SPE method” (accuracy: 91.2–104.8%, precision: 2.5–5.8 (CV%)) [23]. The between-run accuracy of the “LC for PPT method” was 99.4–104.9%. The between-run precision of the “LC for PPT method” was 1.8–8.3 (CV%). The between-run accuracy and precision of the “LC for PPT method” were also better than in the “LC for SPE method” (accuracy: 95.3–108.6%, precision: 3.5–13.9 (CV%)) [23]. The accuracy and precision of the “LC for PPT method” were acceptable. Because of these improved results, new TRAM-34 LC-MS/MS analytical method is accurate and precise. In addition, the improvements of this analytical method directly showed that the strategy of visualized MEs is feasible and useful.

**Table 3:** The accuracy and precision of within-run and between-run of TRAM-34 in rat plasma (n = 6).

	Nominal concentration (ng/ml)	Within-run			Between-run		
		Mean concentration (ng/ml) ± s.d.	Precision (CV, %)	Accuracy (%)	Mean concentrati on (ng/ml) ± s.d.	Precision (CV, %)	Accuracy (%)
LLOQ	1	1.0 ± 0.1	6.9	96.2	1.0 ± 0.1	8.3	99.4
LQC	3	3.1 ± 0.1	2.0	104.7	3.0 ± 0.2	6.5	101.6
MQC	450	467.2 ± 6.2	1.3	103.8	469.0 ± 8.4	1.8	104.2
HQC	800	808.3 ± 10.0	1.2	101.0	839.5 ± 39.0	4.6	104.9

## Conclusion

In this study, we showed the benefits of using visualized MEs in the development of LC-MS/MS methods. The application of visualized MEs could not only predict the ion-suppression zone but also helps in selecting an appropriate (1) mobile phase (2) column, (3) needle wash solvent for the residue of analyte and phospholipids, and (4) evaluate the clean-up efficiency of the sample preparation. The improved TRAM-34 LC-MS/MS method was precise and accurate. This study described the integrative view of the qualitative, quantitative, and visualized MEs for TRAM-34. This is an appropriate model to decipher the complexity of MEs by examining three kinds of MEs. Visualized MEs indeed provided useful information about the LC-MS/MS method development, validation, and improvement.

## Supporting Information

S1 Fig Product ion scan of *m/z* 104.1 and 184.1. (A) The m/z 104.1 → 104.1 transition was tuned after this spectrum was acquired. It represents the existence and abundance of LPCs in the plasma. (B) The *m/z* 184.1 → 184.1 transition was tuned after this spectrum was acquired. It represents the existence and abundance of LPCs and PCs in the plasma.
(TIF)

S2 Fig Visualized elution patterns of phospholipids and IS in in-source multiple-reaction monitoring (IS-MRM) channels. Elution of IS in three different mobile phases. There was no significant interference by coeluted phospholipids with IS.
(TIF)

S3 Fig Qualitative matrix effects (assessment by postcolumn infusion) for IS. Qualitative matrix effects of IS in three different mobile phases. The number of dips was different under different relative conditions of IS. This indicated that the matrix effects were compound-dependent.
(TIF)

S4 Fig Qualitative matrix effects for TRAM-34 in the early elution. Comparison of IS-MRM transitions and qualitative matrix effects in the early elution. The coincidence of the peaks in IS-MRM transitions and dips in the qualitative matrix effects suggested that phospholipids might be responsible for ion suppression in the early elution.
(TIF)

S5 Fig Lipid species profiles. There were 102 species of LPCs, PCs, and SMs identified by LipidView (AB Sciex).
(TIF)

S6 Fig Spectrum of the confirmed and common species in Q1 scan. Q1 full scan raw data were imported into LipidView software, and the lipid species were identified. Many peaks could not be identified by the LipidView software, which represents that phospholipids might not the only source to cause matrix effects in the LC-MS/MS bioanalysis. The LC conditions were as follows: mobile phase A, was 0.2% FA in water; mobile phase B, 0.2% FA in ACN/MeOH (1:1 v/v), 0.2% FA in MeOH, or 0.2% FA in ACN. The flow rate was 400 μL/min. The gradient elution was as follows: mobile phase A: 0–1 min, 70–30%; 1–1.5 min, 30–0%; 1.5–4.5 min, 0%; and 4.51–7.5 min, 70% (LC for SPE method).
(TIF)

S7 Fig Identified tentative phospholipids species in the chromatogram of MeOH/ACN 50/50. (A) Five tentative phospholipids (*m/z* 496.5 = LPC 16:0, *m/z* 520.5 = LPC 18:2, *m/z* 524.5 = LPC 18:0, *m/z* 542.4 = LPC 20:5, and *m/z* 747.8 = SM 36:1;3) were identified at RT for 4.0–5.0 min. They may be responsible for ion suppression in that region. In addition, the rest of un-identified peaks might also be the potential candidates for the ion suppression in “LC for SPE method”. (B) Comparison of Q1 and precursor ion scans of *m/z* 184.1 by RT, *m/z*, and intensity. The RT interval was 4.0–5.0 min, and the *m/z* range was 400–1000. Q1 full scan presented more possible candidates as causing the matrix effects than the precursor ion scan of *m/z* 184.1.
(TIF)

S8 Fig Visualized matrix effects in the improved TRAM-34 LC-MS/MS analytical method. The RT of TRAM-34 in “LC for PPT method” was 3.28 ± 0.2 min. The elution time of TRAM-34 and PCs would slightly shift at the same time. The stability of RT was relatively stable. Elution sequence of phospholipids and TRAM-34 in 100% ACN was unchanged compared to Fig 3C. The late eluted phospholipids are only visible in *m/z* 184.1 → 184.1 transition and were regarded as PCs. After SPE processing, the elution of PCs (Fig 3C) was smaller than after PPT processing (Fig. S8). These observations indicated that the late-eluted PCs were efficiently removed by SPE. The setting of IS-MRM was also useful to assess the extent of clean-up of the sample during preparation.
(TIF)

S9 Fig Ability of solvent to wash TRAM-34 phospholipid residue. (A) IPA was the best wash solvent for TRAM-34. ACN was the worst. (B) MeOH was the best wash solvent for the phospholipids. ACN was the worst.
(TIF)
