# Supplementary figures and images for: Using Visualized Matrix Effects to Develop and Improve LC-MS/MS Bioanalytical Methods, Taking TRAM-34 as an Example
Source: PLoS One. 2015 Apr 24;10(4):e0118818. doi: 10.1371/journal.pone.0118818 (PMC4409340; doi:10.1371/journal.pone.0118818)

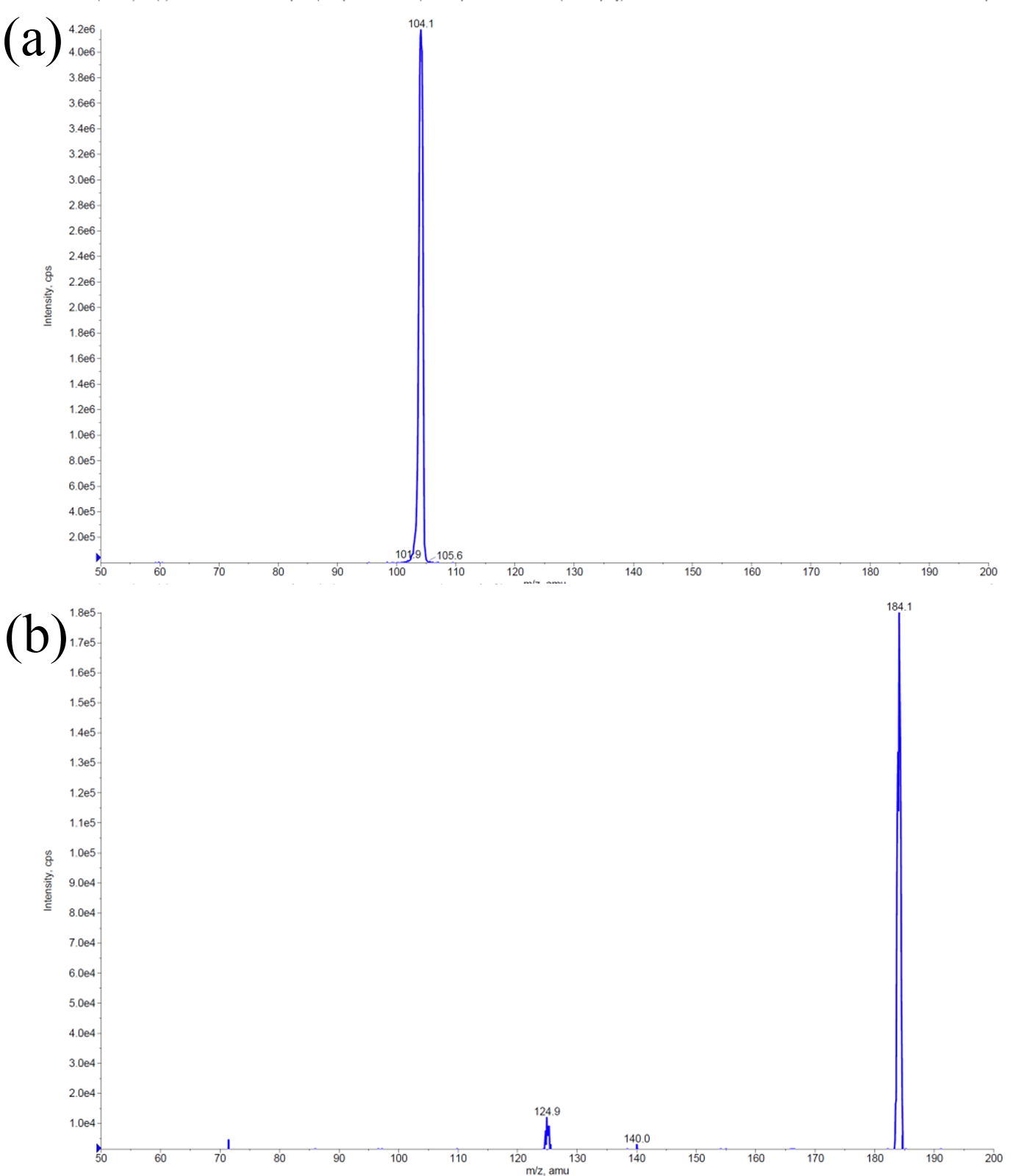

Supplement: S1 Fig — (A) The m/z 104.1 → 104.1 transition was tuned after this spectrum was acquired. It represents the existence and abundance of LPCs in the plasma. (B) The m/z 184.1 → 184.1 transition was tuned after this spectrum was acquired. It represents the existence and abundance of LPCs and PCs in the plasma. (TIF) [file pone.0118818.s001.tif]

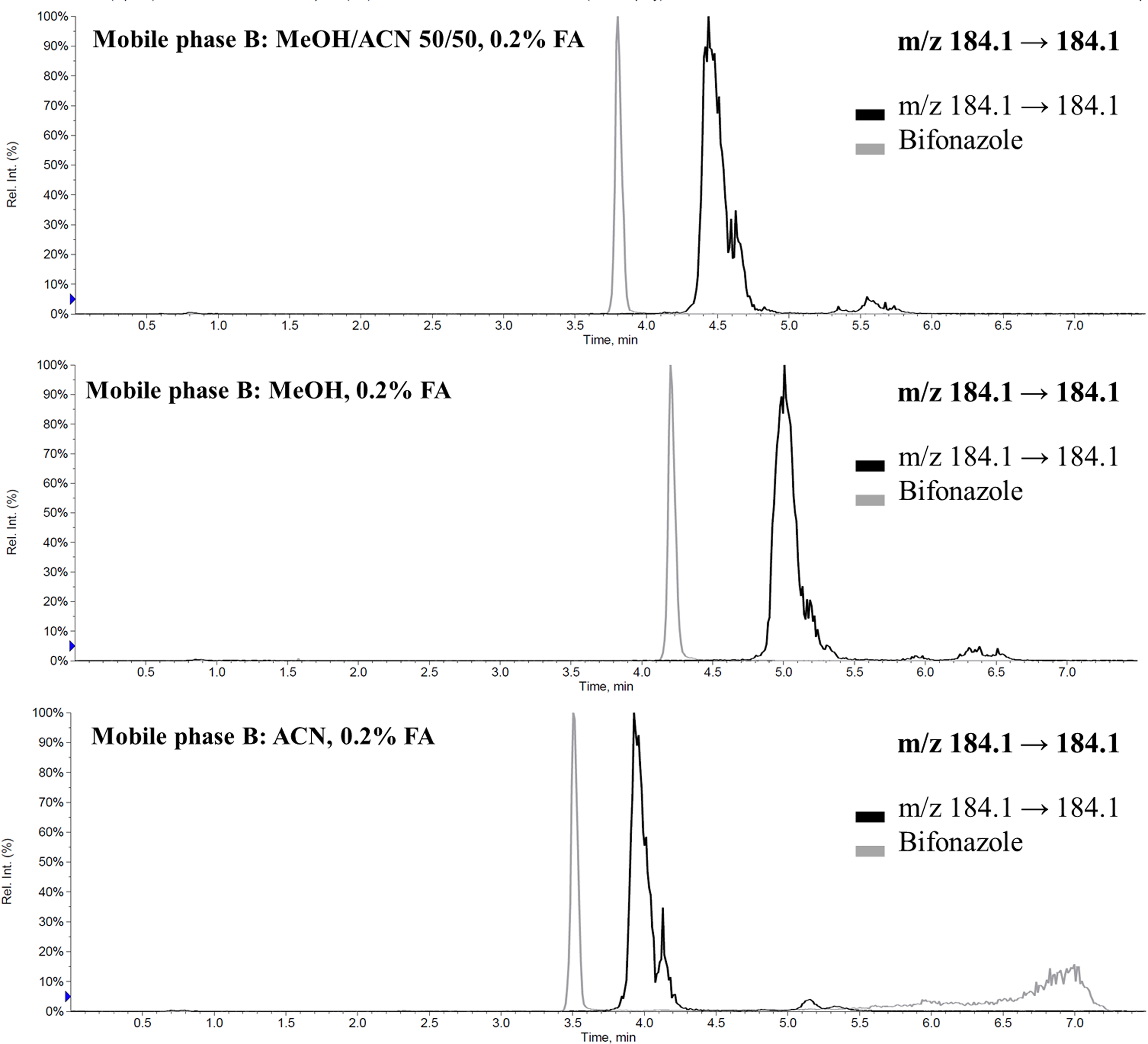

Supplement: S2 Fig — Elution of IS in three different mobile phases. There was no significant interference by coeluted phospholipids with IS. (TIF) [file pone.0118818.s002.tif]

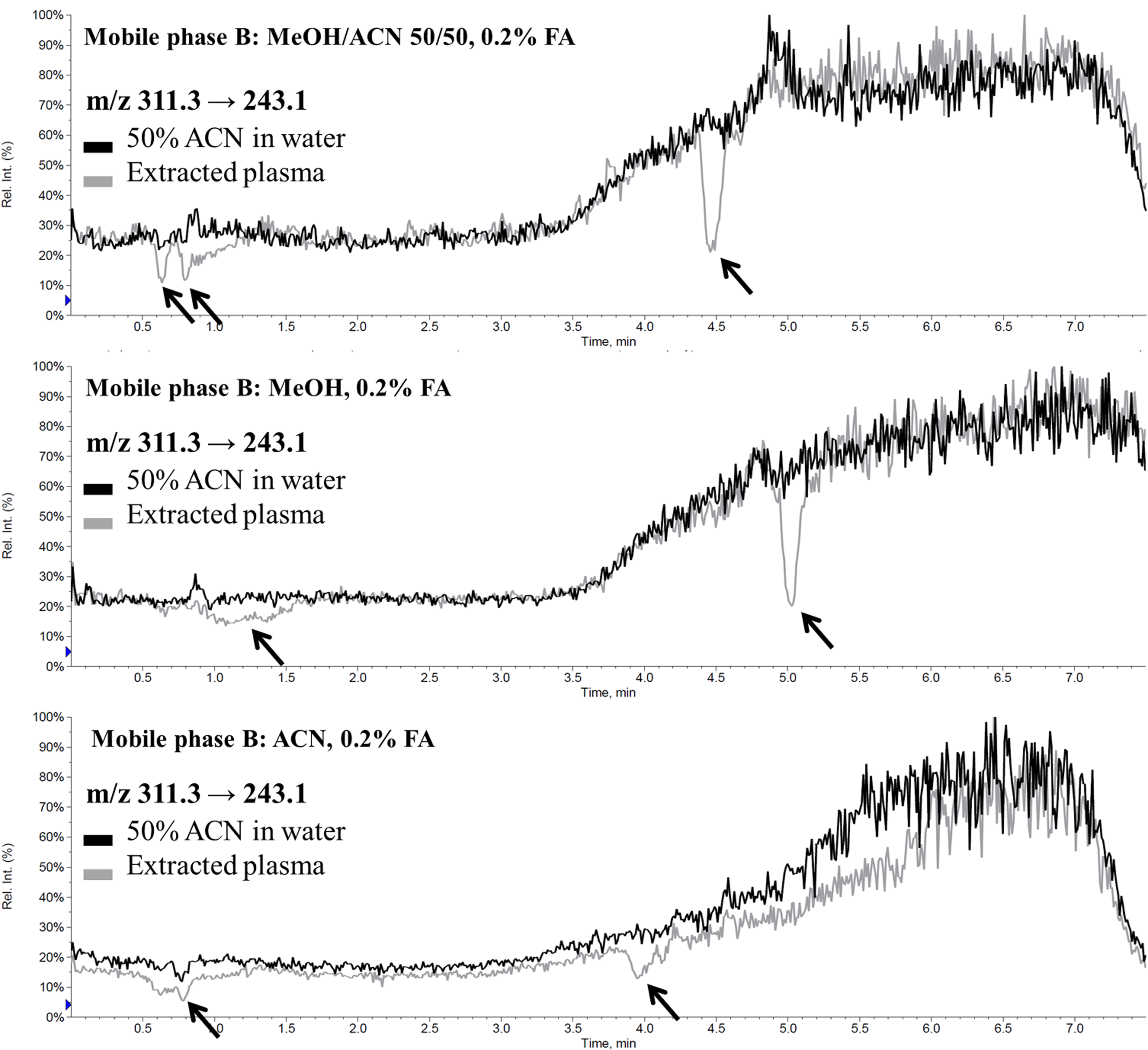

Supplement: S3 Fig — Qualitative matrix effects of IS in three different mobile phases. The number of dips was different under different relative conditions of IS. This indicated that the matrix effects were compound-dependent. (TIF) [file pone.0118818.s003.tif]

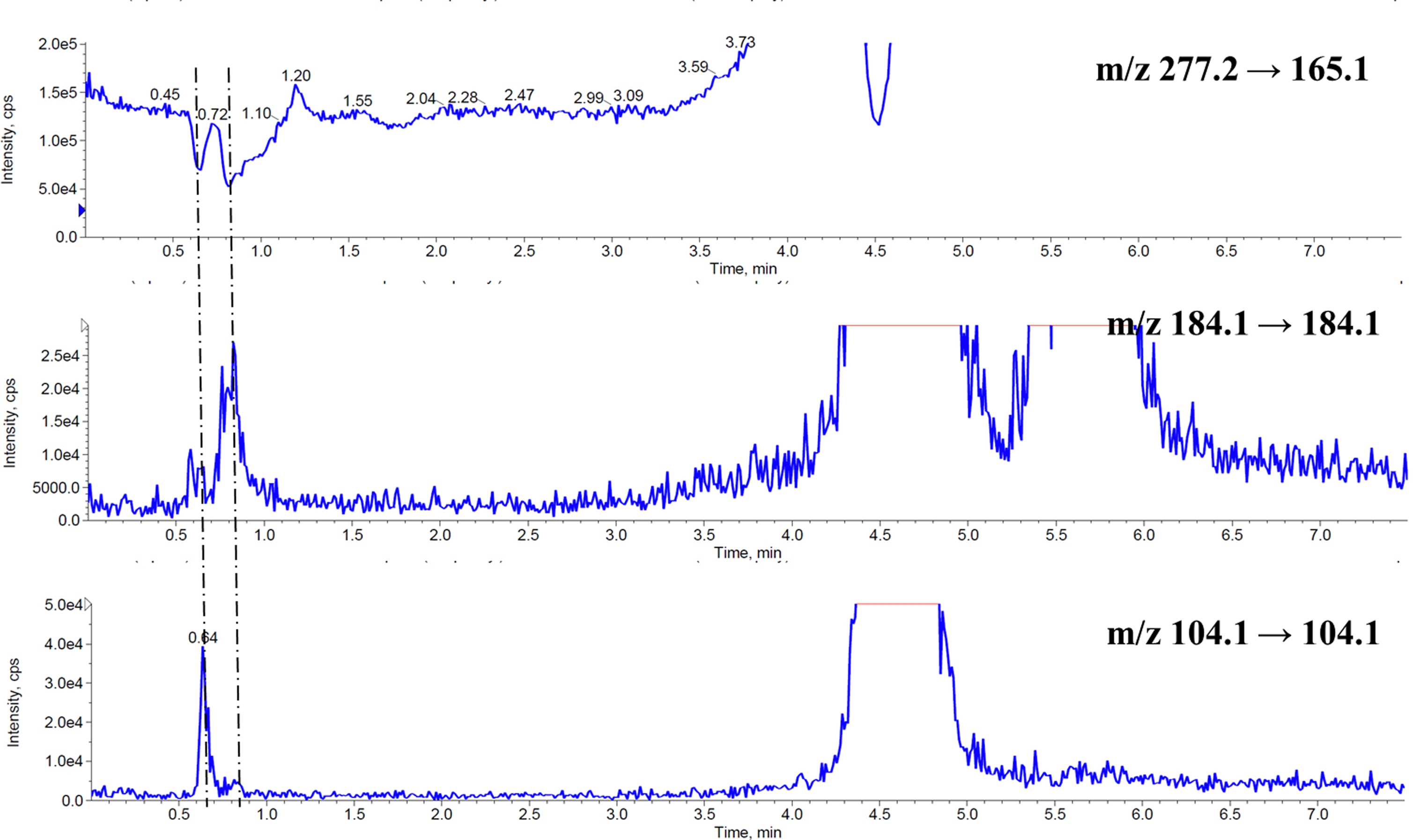

Supplement: S4 Fig — Comparison of IS-MRM transitions and qualitative matrix effects in the early elution. The coincidence of the peaks in IS-MRM transitions and dips in the qualitative matrix effects suggested that phospholipids might be responsible for ion suppression in the early elution. (TIF) [file pone.0118818.s004.tif]

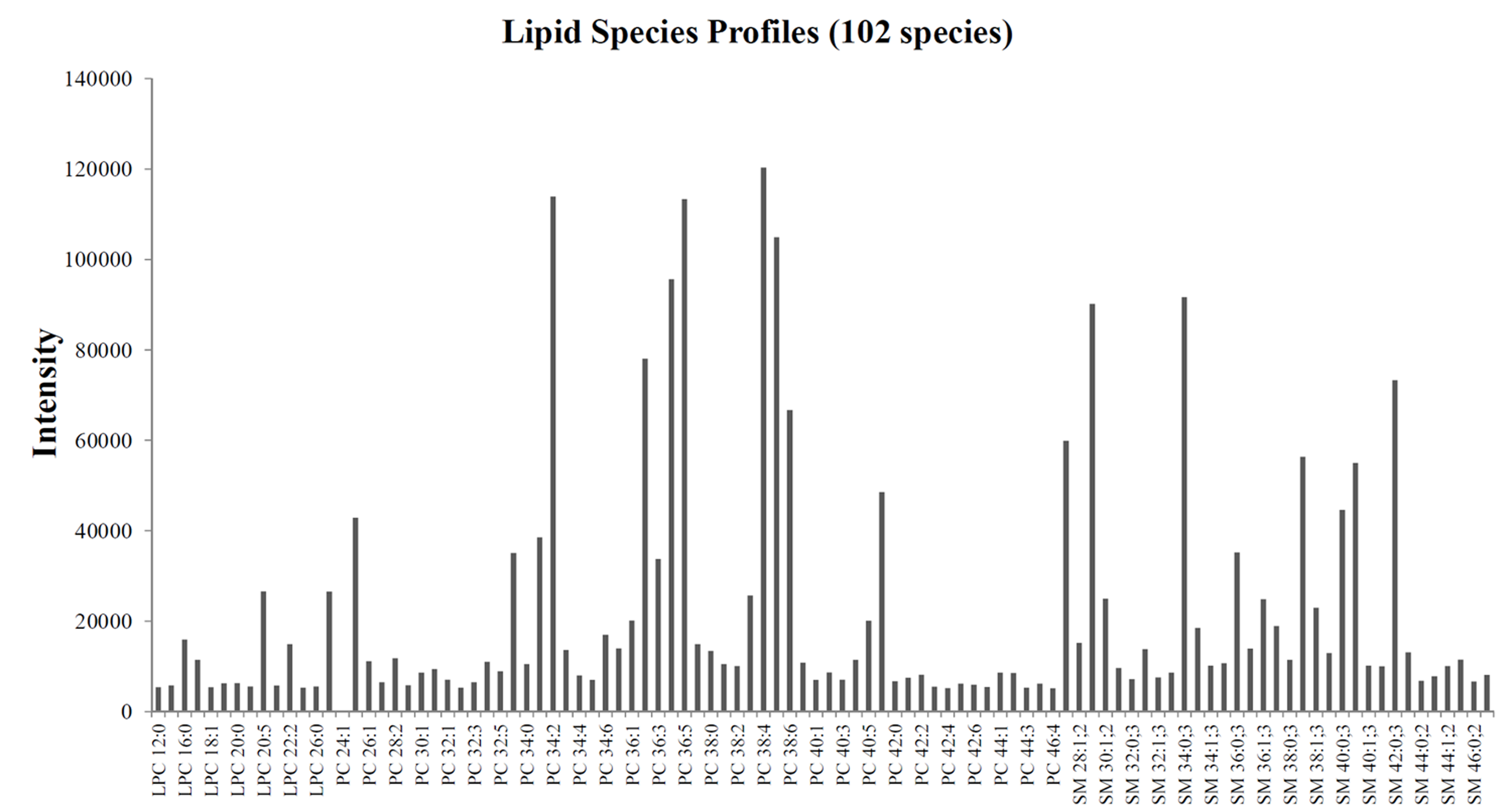

Supplement: S5 Fig — There were 102 species of LPCs, PCs, and SMs identified by LipidView (AB Sciex). (TIF) [file pone.0118818.s005.tif]

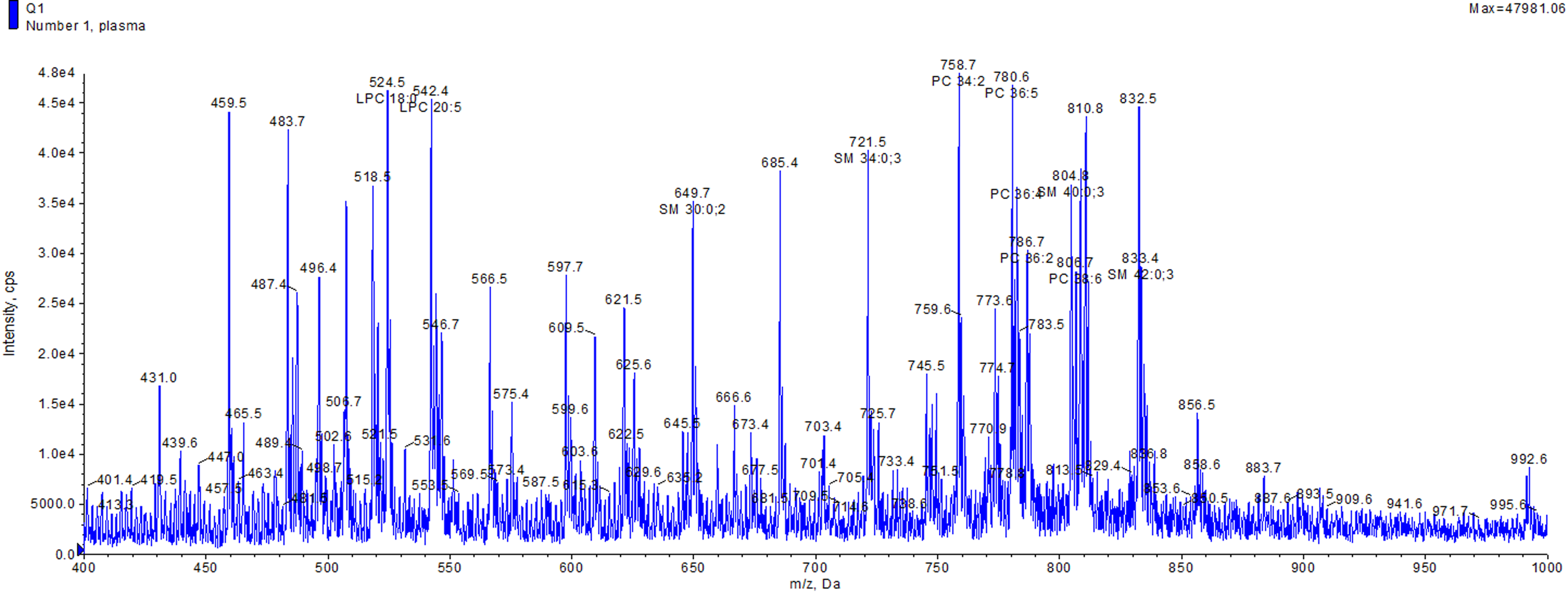

Supplement: S6 Fig — Q1 full scan raw data were imported into LipidView software, and the lipid species were identified. Many peaks could not be identified by the LipidView software, which represents that phospholipids might not the only source to cause matrix effects in the LC-MS/MS bioanalysis. The LC conditions were as follows: mobile phase A, was 0.2% FA in water; mobile phase B, 0.2% FA in ACN/MeOH (1:1 v/v), 0.2% FA in MeOH, or 0.2% FA in ACN. The flow rate was 400 μL/min. The gradient elution was as follows: mobile phase A: 0–1 min, 70–30%; 1–1.5 min, 30–0%; 1.5–4.5 min, 0%; and 4.51–7.5 min, 70% (LC for SPE method). (TIF) [file pone.0118818.s006.tif]

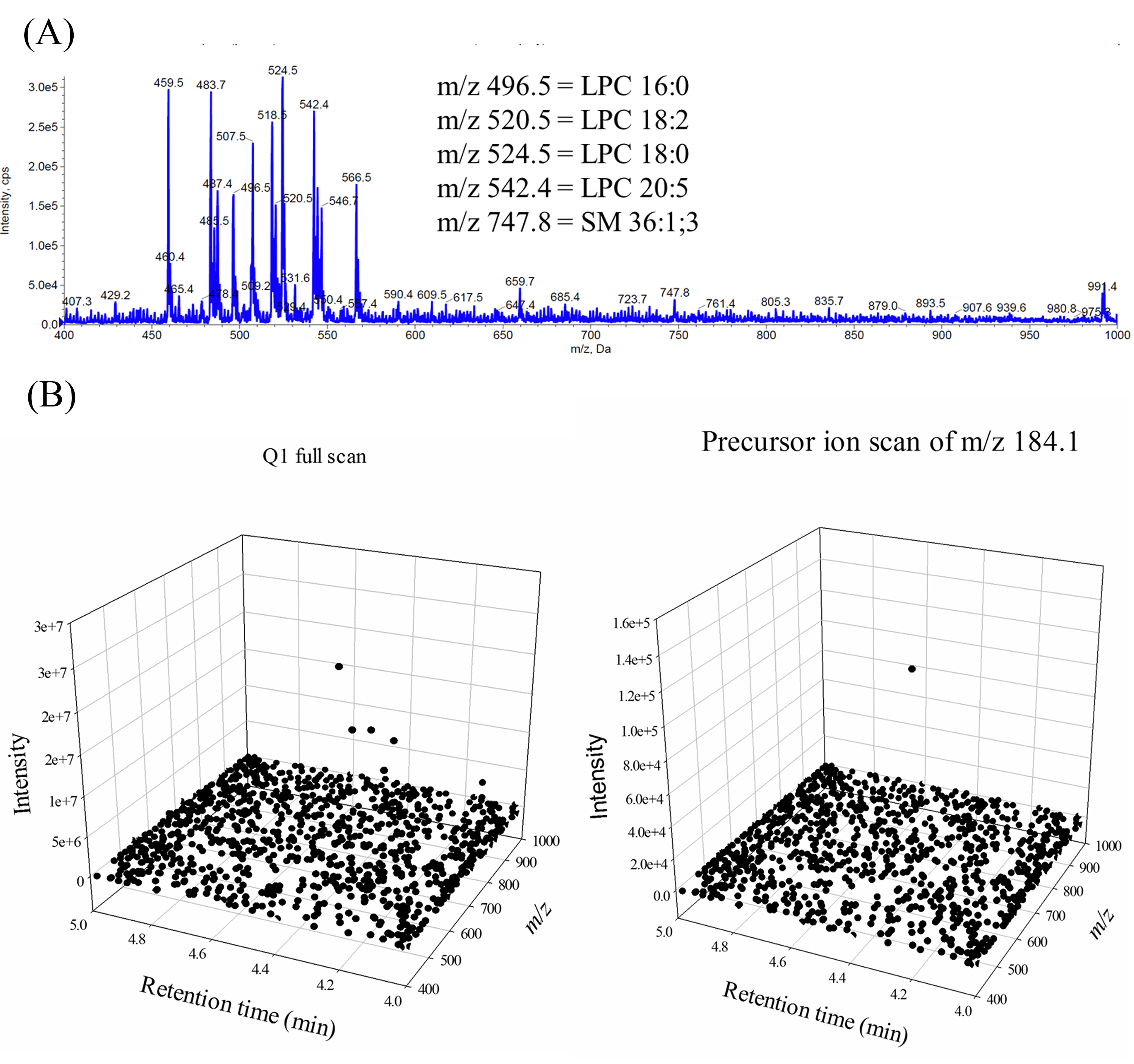

Supplement: S7 Fig — (A) Five tentative phospholipids (m/z 496.5 = LPC 16:0, m/z 520.5 = LPC 18:2, m/z 524.5 = LPC 18:0, m/z 542.4 = LPC 20:5, and m/z 747.8 = SM 36:1;3) were identified at RT for 4.0–5.0 min. They may be responsible for ion suppression in that region. In addition, the rest of un-identified peaks might also be the potential candidates for the ion suppression in “LC for SPE method”. (B) Comparison of Q1 and precursor ion scans of m/z 184.1 by RT, m/z, and intensity. The RT interval was 4.0–5.0 min, and the m/z range was 400–1000. Q1 full scan presented more possible candidates as causing the matrix effects than the precursor ion scan of m/z 184.1. (TIF) [file pone.0118818.s007.tif]

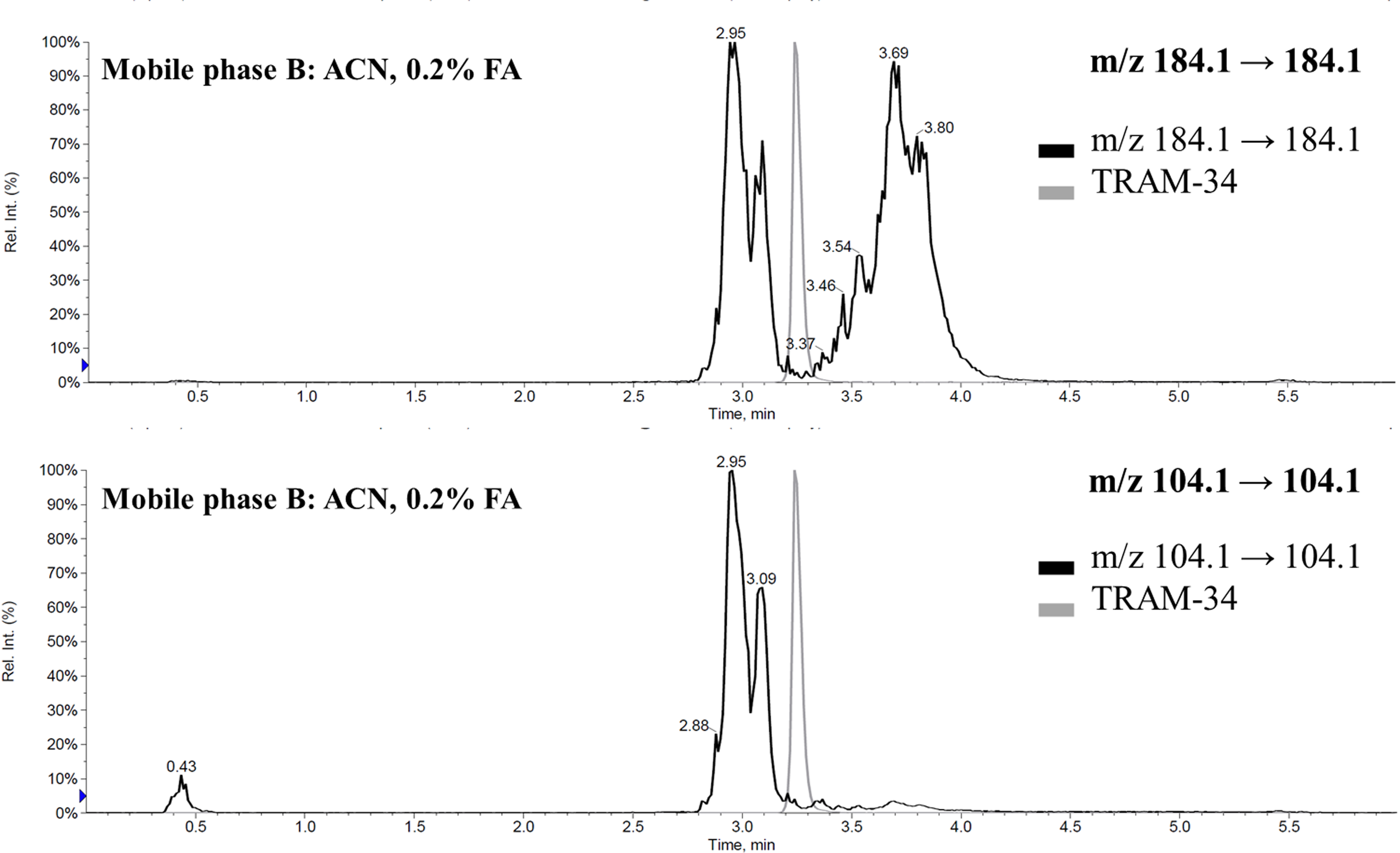

Supplement: S8 Fig — The RT of TRAM-34 in “LC for PPT method” was 3.28 ± 0.2 min. The elution time of TRAM-34 and PCs would slightly shift at the same time. The stability of RT was relatively stable. Elution sequence of phospholipids and TRAM-34 in 100% ACN was unchanged compared to Fig 3C. The late eluted phospholipids are only visible in m/z 184.1 → 184.1 transition and were regarded as PCs. After SPE processing, the elution of PCs (Fig 3C) was smaller than after PPT processing (Fig. S8). These observations indicated that the late-eluted PCs were efficiently removed by SPE. The setting of IS-MRM was also useful to assess the extent of clean-up of the sample during preparation. (TIF) [file pone.0118818.s008.tif]

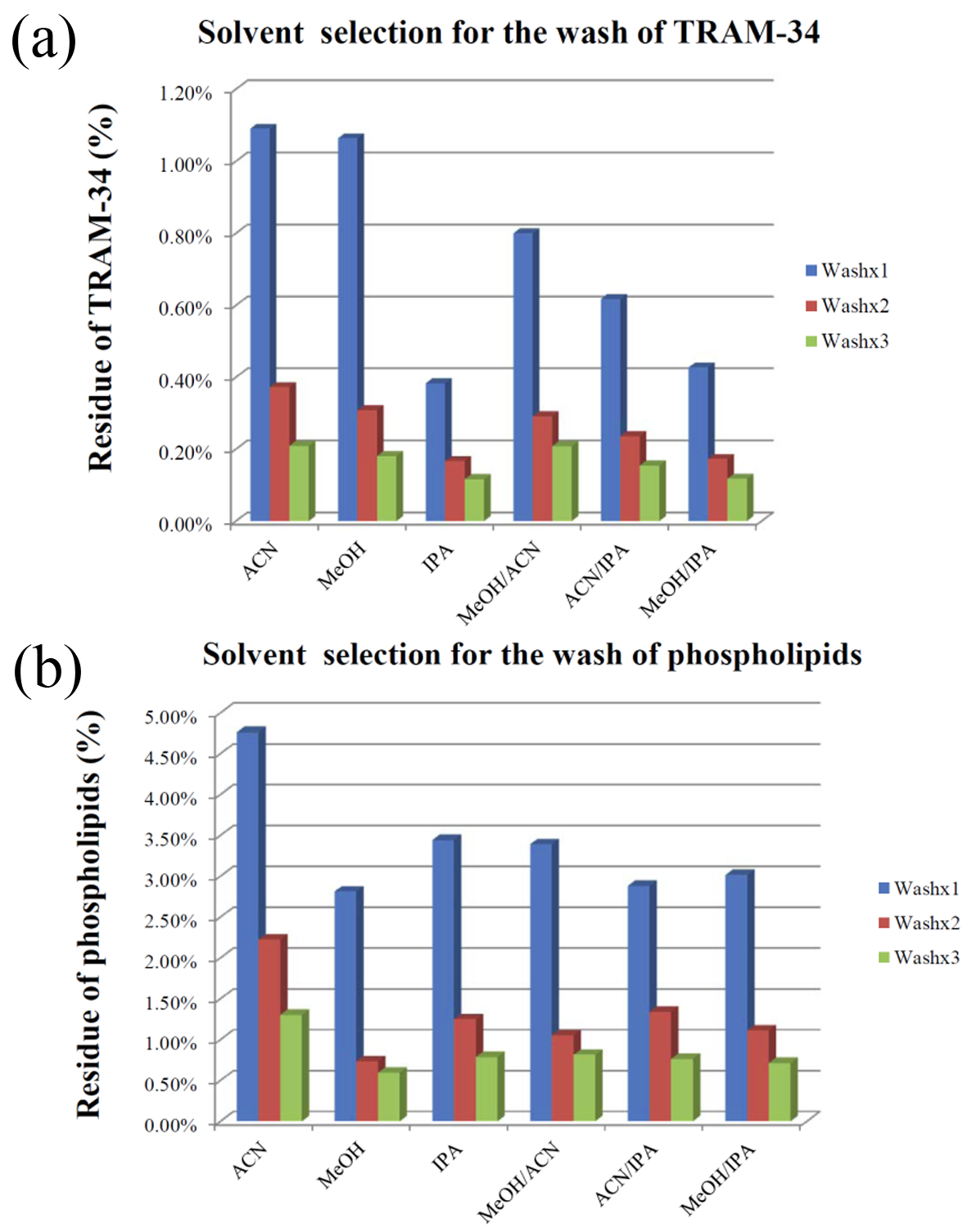

Supplement: S9 Fig — (A) IPA was the best wash solvent for TRAM-34. ACN was the worst. (B) MeOH was the best wash solvent for the phospholipids. ACN was the worst. (TIF) [file pone.0118818.s009.tif]
